# Insight into Covid Associated Mucormycosis: A Prospective Study

**DOI:** 10.22038/ijorl.2024.78990.3662

**Published:** 2025

**Authors:** Avinash-Shekhar Jaiswal, Aanchal Kakkar, Kavneet Kaur, Alok Thakar, Kapil Sikka, Hitesh Verma

**Affiliations:** 1 *Department of Otorhinolaryngology and Head & Neck Surgery, All India Institute of Medical Sciences, New Delhi, India.*; 2 *Department of Pathology, All India Institute of Medical Sciences, New Delhi, India.*

**Keywords:** COVID-19, SARS-CoV-2, Mucormycosis, Angiotensin-Converting Enzyme 2, Transmembrane serine protease 2

## Abstract

**Introduction:**

The notable increase in cases of rhino-orbito-cerebral Mucormycosis during the COVID pandemic is alarming. Both share a common route of entry, the nasal mucosa, leading to speculation about whether similar receptors play a role in both diseases. We aim to compare the expression of ACE2 and TMPRSS2 in the nasal and paranasal sinus tissues among patients with COVID-19-associated Mucormycosis (CAM), COVID-19-negative mucormycosis (CNM), and healthy individuals.

**Materials and Methods:**

This prospective study included patients with CAM, CNM, and healthy individuals who underwent surgical management. Immunohistochemistry was performed in the sino-nasal tissue to detect the presence of ACE2 and TMPRSS2 receptors. The level was compared among the three groups.

**Results:**

The study encompassed 44 patients with CAN, 20 with CNM, and ten healthy individuals. ACE2 positivity was seen only in the apical cilia, with no significant difference among the groups (p=0.6). In contrast, TMPRSS2 positivity was seen in the cytoplasm and nucleus of epithelium and submucosal glands in addition to apical cilia. TMPRSS2 was increasingly expressed in patients with CAM compared to CNM (p=0.009) and the healthy group (p=0.002).

**Conclusion:**

The expression of TMPRSS2 receptors is elevated in patients with COVID-19-associated mucormycosis with no significant change in the expression of ACE2 receptors. This finding could account for the heightened susceptibility to infection by SARS-CoV-2 and the subsequent immune dysregulation, providing a fertile ground for Mucorales co-infection.

## Introduction

Mucormycosis refers to the dreaded fungal infection caused by the organisms of the order Mucorales, which includes Rhizopus spp, Mucor spp, Lichtheimia spp, Rhizomucor spp, Cunninghamella spp, Apophysomyces spp, and Saksanaea spp (1-3). These are ubiquitous filamentous fungi causing angioinvasive infection. The population at risk for mucormycosis has expanded over time from those with lung malignancy to uncontrolled diabetes mellitus. Other groups who are at risk include recipients of solid organ and haematopoietic stem cell transplant patients undergoing chemotherapy and immunotherapy treatments (1,4,5). The global pandemic due to COVID-19 caused by severe acute respiratory syndrome coronavirus (SARS-CoV-2) is the new addition to the list. The significant rise in COVID-19-associated mucormycosis (CAM) is alarming, considering the high morbidity and mortality associated with rhino-orbital cerebral mucormycosis (ROCM) (1,6-9). 

The main route of infection in cases with ROCM remains the inhalation of sporangiospores, leading to the involvement of the mucosa of the nose and paranasal sinuses. Mucorales-specific T-cells, tissue macrophages, and neutrophils are adequate to generate an effective reaction against inhaled sporangiospores and hyphal elements of the Mucorales (10,11). 

The nasal epithelium also serves as the major route of entry of SARS-CoV-2 (12). The virus utilizes spike (S) protein anchored in its envelope for binding to the epithelial cells. Viral infection is initiated after binding the S protein’s receptor binding domain (RBD) to the host cell’s angiotensin-converting enzyme 2 (ACE2). 

The virus also depends on the host protease, transmembrane serine protease 2 (TMPRSS2), which helps cleavage the S protein. TMPRSS2 is responsible for the fusion of the virus with the host cell membrane before it enters the host cell. Without TMPRSS2, SARS-CoV-2 can also use other proteases, such as cathepsin B/L, for viral fusion. However, TMPRSS2 seems to be the predominant one responsible for this priming (13,14). 

The infection leads to local immune dysregulation, reducing the numbers of T-lymphocytes, neutrophils, and macrophages, resulting in an increased propensity for secondary fungal infections (11,15). The increased presence of ACE2 and TMPRSS2 in the tissues increases their susceptibility to infection by SARS-CoV-2 (14,16). This increased susceptibility may also be responsible for the local immune dysregulation, which makes these sites more vulnerable to secondary fungal infections like mucormycosis. It could be one of the contributing factors to an increased prevalence of mucormycosis in patients with COVID-19. 

We conducted a prospective study to compare the expression of ACE2 and TMPRSS2 in the nasal and paranasal sinus tissues among patients with COVID-19-associated mucormycosis, COVID-19-negative mucormycosis (CNM), and healthy individuals.

## Materials and Methods

This prospective study was undertaken in the Department of Otorhinolaryngology and Head-Neck Surgery from August 2021 to May 2023 after approval from the Institute Ethics Committee (IEC-559/06.08.2021, RP-17/2021). The study included patients of COVID-19-associated rhino-orbital-cerebral mucormycosis who were diagnosed based on the presence of aseptate hyphae on histopathological and/or direct Potassium hydroxide mount of the sino-nasal tissue. All the participants gave informed written consent for the study. The patients who underwent surgical debridement in the department during the study period were included and labelled as group A. 

The archives of the Department of Pathology were used to include patients of COVID-19 negative rhino-orbital-cerebral mucormycosis who underwent surgical debridement before the onset of the COVID-19 pandemic and were designated as group B. 

The control group, group C, included healthy tissues from patients who underwent surgical procedures of the nose and paranasal sinuses for diseases apart from mucormycosis. The debrided tissue taken from nasal mucosa and/or paranasal sinus during biopsy or surgery was collected in 10% formalin and sent to the Department of Pathology to estimate ACE2 and TMPRSS2 levels by immunohistochemistry.

2.1 Sample processing and estimation of ACE2 and TMPRSS2 expression:

Histopathological examination – The biopsy specimens were fixed in 10% formalin and underwent further processing in the Department of Pathology. Paraffin-embedded tissue blocks were prepared to create stained haematoxylin and eosin (H&E) slides. Immunohistochemistry (IHC) was performed to demonstrate ACE2 and TMPRSS2 within the viable tissue sections. Representative sections from the tissue blocks were cut and overlaid on poly-L-lysine-coated slides. Antigen retrieval was done after sequential deparaffinization, followed by rehydration. Slides were treated with methanol and 4% hydrogen peroxide to reduce endogenous peroxidase activity. 

Adequately diluted primary antibody was applied to the sections. Sections were kept overnight and incubated at 4 degrees Celsius in a humid chamber. 

A primary antibody amplifier was applied to the slides, followed by incubation at room temperature for 10 minutes and HRP polymer incubation for 10 minutes. 

The slides were then rinsed for the addition of substrate chromogen solution. The slides were further incubated at room temperature for 5-10 minutes. Counterstaining with haematoxylin was done, and slides were mounted with DPX. Proper positive and negative controls were implemented. 

2.2 Outcome measures: ACE2 and TMPRSS2 expression level and distribution in diseased and healthy tissues.

2.3 Statistical analysis: Data were described as median and mean. The chi-square and Mann-Whitney tests were used to compare sex and age. The levels of ACE2 and TMPRSS2 were compared among the group in pairs using the chi-square test. Stata/MP 16 was used for statistical analysis. The data was considered to have significance where the p-value was less than 0.05.

## Results

The study included 44 patients in group A with COVID-19-associated mucormycosis, 20 in group B with COVID-19-negative mucormycosis, and 10 in group C comprising healthy controls. 

**Fig 1:**
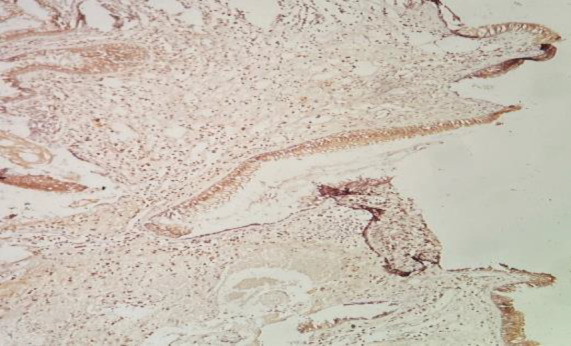
Photomicrograph of the sino-nasal tissue at 10x stained with antibodies to ACE2 receptors showing positive staining of apical cilia

The clinical, demographic, and laboratory characteristics are described in Table 1. There was no significant difference among the groups in the baseline distribution of age and sex. ACE2 positivity was seen only in the apical cilia of the respiratory epithelium (Figure 1). 

**Fig 2:**
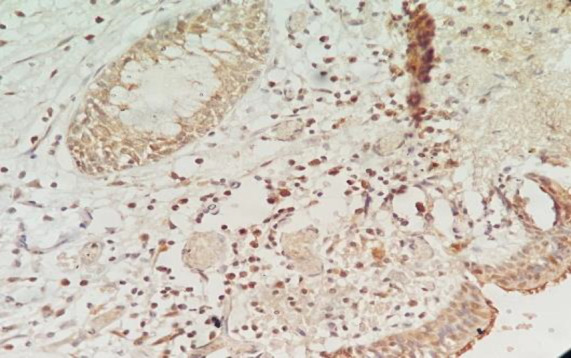
Photomicrograph of the sino-nasal tissue at 40x stained with antibodies to TMPRSS2 receptors showing positive staining of cytoplasm and nucleus of both epithelium and submucosal glands

**Fig 3:**
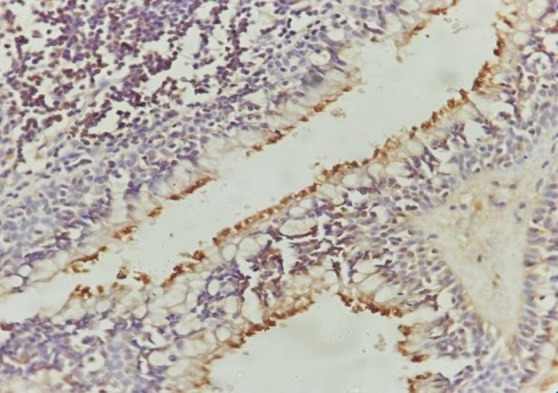
Photomicrograph of the sino-nasal tissue at 40x stained with antibodies to TMPRSS2 receptors showing positive staining of apical cilia of the respiratory epithelium

There were no significant differences between the groups in the distribution of ACE2 positivity. The p-value for Group A versus Group B was 0.63, Group A versus Group C was 0.316, and Group B versus Group C was 0.41. In contrast to ACE2, TMPRSS2 positivity was present in the cytoplasm and nucleus of both epithelium and submucosal glands (Figure 2), in addition to the presence on the apical cilia of the respiratory epithelium (Figure 3). 

The difference in the TMPRSS2 positivity was statistically significant between the A and B groups (p=0.009) as well as the A and C groups (p=0.002). No significant difference was observed between groups B and C (p=0.331). Additionally, we found that the pattern of positivity of TMPRSS2 was different in groups A and B. In group A, TMPRSS2 positivity was predominantly seen in the cytoplasm of the respiratory epithelium and submucosal glands (39/44), whereas, in group B, the predominant positivity was seen in the nucleus of the respiratory epithelium and submucosal glands (10/14). This difference was determined to be statistically significant (p<0.001). In group C, 1/5 of the cases had nuclear positivity for TMPRSS2. Group B had a significantly increased nuclear positivity for TMPRSS2 compared to group C (p=0.017). No significant difference was observed between groups A and C in the nuclear positivity for TMPRSS2 (p=0.665).

**Table 1:** Clinico-demographical characteristics of the study groups

	**Group A**	**Group B**	**Group C**	**p-value**
**Total number**	44	20	10	
**Age (in years)**				0.9
Mean	50.4	45	49.7	
Range	26 to 71	29 to 67	30 to 68	
**Sex**				0.27
Male	32	13	8	
Female	12	7	2	
**ACE2**				0.79
Positive	20	9	6	
Negative	24	11	4	
**TMPRSS2**				**<0.001**
Positive in the epithelium and/or submucosal glands	42	14	5	
Positive only in apical cilia	2	0	4	
Negative	0	6	1	

## Discussion

For decades, viral infections have been linked to secondary fungal co-infection. The association has been so great that certain fungal infections have even been used for viral-defining infections - such as pneumocystis pneumonia and cryptococcosis for AIDS (17,18). The link between invasive fungal diseases with cytomegalovirus infections in patients who have undergone stem-cell transplants and invasive aspergillosis in cases of Influenza is well known (19,20). Mucormycosis has been proven to be significantly associated with cases of COVID-19 (21,22). Mucorales have predominantly infected individuals with localized or systemic immune dysregulation, allowing them to escape mononuclear and polymorphonuclear phagocytes, which otherwise would have eliminated the fungal spores and hyphae through oxidative and non-oxidative mechanisms. Morton et al. have summarized the risk factors for invasive fungal disease in COVID-19 patients. A breakdown in the innate anti-fungal immunity caused by disruption of the respiratory epithelium, decreased mucociliary clearance, impaired phagocytosis, and immune exhaustion play a combined role in predisposing COVID-19 patients to secondary fungal infection (23).

Lee et al. have shown the presence of ACE2 receptors in the muti-ciliated epithelial cells of the airway. They have also found that ACE2 is localized to the motile cilia of the inferior turbinate, uncinate process, and ethmoid sinuses (24). 

The authors have also shown that the ciliary expression of ACE2 remains unchanged with age, sex, or smoking. Our findings were consistent with their observation that ACE receptors are distributed predominantly in the apical cilia of nasal epithelium. The presence of ACE2 provides the necessary molecular component to allow the entry of SARS-CoV-2 within the respiratory epithelium of the nasal cavity. However, there was no significant difference in the distribution of ACE receptors among cases with CAM, CNM, and healthy controls. Increased expression of ACE receptors does not determine the susceptibility of patients to SARS-CoV-2 in this patient group. 

In contrast, TMPRSS2 was significantly increased in patients with CAM compared to controls and CNM. The higher expression of TMPRSS2 has been associated with augmented S-protein priming, leading to increased susceptibility for SARS-CoV-2 infection in the general population (12). 

Hence, the increased expression of TMPRSS2 may be one of the factors responsible for the increased susceptibility to COVID-19 infection, leading to the necessary immune dysregulation for Mucorales co-infection in patients with CAM. Also, there is no significant difference in the expression of TMPRSS2 between CNM and controls, implying that the receptors are associated with COVID-19 and not with mucormycosis per se.

Our study explores the role of ACE2 and TMPRSS2 expression in patients with CAM. However, we have not looked at the factors increasing the TMPRSS2 expression in these patients. 

Also, the significance of differential expression of TMPRSS2 within the cytoplasm and nucleus of epithelium and glands in patients with CAM and CNM has yet to be discovered.

## Conclusion

An abrupt increase in the cases of mucormycosis has been noted during the pandemic caused by SARS-CoV-2. We found that the expression of TMPRSS2 receptors is increased in patients with COVID-19-associated mucormycosis with no significant change in the expression of ACE2 receptors. The heightened susceptibility to infection by SARS-CoV-2 may be secondary to increased TMPRSS2 receptor expression, which causes subsequent immune dysregulation and provides a fertile ground for Mucorales co-infection. 
